# Statistical modeling and evaluation of the impact of multiplicity classification thresholds on the COVID-19 pool testing accuracy

**DOI:** 10.1371/journal.pone.0283874

**Published:** 2023-07-26

**Authors:** Omar De La Cruz Cabrera, Razan Alsehibani

**Affiliations:** Department of Mathematical Sciences, Kent State University, Kent, OH, United States of America; Universidad de Valladolid, SPAIN

## Abstract

Prior research on pool testing focus on developing testing methods with the main objective of reducing the total number of tests. However, pool testing can also be used to improve the accuracy of the testing process. The objective of this paper is to improve the accuracy of pool testing using the same number of tests as that of individual testing taking into consideration the probability of testing errors and pool multiplicity classification thresholds. Statistical models are developed to evaluate the impact of pool multiplicity classiffcation thresholds on pool testing accuracy using the receiver operating characteristic (ROC) curve and the area under the curve (AUC). The findings indicate that under certain conditions, pool testing multiplicity yields superior testing accuracy compared to individual testing without additional cost. The results reveal that selecting the multiplicity classification threshold is a critical factor in improving the pool testing accuracy and show that the lower the prevalence level the higher the gains in accuracy using multiplicity pool testing. The findings also indicate that performance can be improved using a batch size that is inversely proportional to the prevalence level. Furthermore, the results indicate that multiplicity pool testing not only improves the testing accuracy but also reduces the total cost of the testing process. Based on the findings, the manufacturer’s test sensitivity has more significant impact on the accuracy of multiplicity pool testing compared to that of manufacturer’s test specificity.

## 1 Introduction

The emergence of COVID-19 resulted in growing severe levels of medical, social, psychological, and economic losses [1–4]. The fast spread of the COVID-19 virus has emphasized the paramount need to test millions of people quickly, efficiently, and effectively in order to curb the proliferation of the disease. Infectious disease testing is a costly process especially when there is a need to quickly test large numbers of people. Testing also might need to be repeated frequently to monitor the spread of the disease. Unlike many other infectious diseases, one challenge facing the combat of COVID-19 is that the majority of cases are asymptomatic individuals who can be contagious [5–7]. Since many of the asymptomatic cases might not be aware of their infection, they need to be quickly identified before they infect others [8].

The economist Robert Dorfman [9] developed a novel pool testing algorithm with the objective of reducing the total number of tests where individual specimens are grouped into a pool to be tested using one test instead of conducting individual testing [9]. If the pool tests negative then all individuals are declared healthy, otherwise a second round of testing is needed. Pool testing is used in several fields to identify “defective” subjects and there is an increasing need for better understanding of not only how to reduce the number of tests but also to increase the accuracy of the pool testing process.

With the emergence of COVID-19, several researchers and practitioners stressed the importance of utilizing pool testing in controlling the spread of the disease [10, 11]. In February 2020, COVID-19 pool testing methods enabled Stanford University’s researchers to quickly identify several positive infections [12]. Pool testing is useful also because negative results can be communicated faster to individuals, since this method reduces the time needed to analyze tests [13]. However, the lack of understanding of how to design an optimal pooling scheme to improve classification accuracy under budget constraints, is hindering screening efforts [14]. Since the Dorfman’s pool testing proposal, researchers introduced several algorithms to implement variations of the original method [15–20].

A main objective of prior research was to improve the efficiency of pool testing by minimizing the number of required tests which consequently reduces the cost of the testing process [21]. Bish et al. [22] develop a robust model based on the Dorfman pool testing method to determine optimal pool size assuming that the perfect test specificity with the objective of reducing the total number of tests. De Wolff et al. [23] and Verdun et al. [24] perform an evaluation of several pool testing methods to identify under what conditions certain algorithms improve testing efficiency. However, there is a need to improve the accuracy of pool testing to increase the effectiveness of the testing process which will not only curb the spread of epidemic disease but also to ultimately reduce the testing costs. The objective of this research is to complement prior research in pool testing by developing models to improve the pool testing accuracy without incurring extra cost, taking into consideration that probability of testing error and pool multiplicity classification threshold.

The contributions of this paper to pool testing research is multi-fold. First, the relevant literature is reviewed to identify research gaps. Next, the multiplicity pool testing method of [20] is extended by including the probability of testing errors and classification thresholds into the modeling process with the objective of improving the pool testing accuracy. The impact of several multiplicity classification thresholds on pool testing specificity and pool testing sensitivity is evaluated analytically and through simulation. The ROC and the AUC methods are employed to evaluate the performance of the proposed models. Then, the impact of batch sizes on pool testing accuracy for specific pool testing multiplicity levels is examined. Finally the effect of the manufacturer’s test sensitivity on the pool testing accuracy is compared to that of the manufacturer test specificity. Thus, the proposed models extend prior research on pool testing (e.g., [14, 20, 25, 26]).

The rest of this paper is organized as follows. Section 2 presents an overview of the classification of pool testing methods. In Section 3, the assumptions of our models are stated and the statistical models are developed. The simulation of our models and the experimental results are discussed in Section 4. Conclusions are presented in Section 5.

## 2 Classification of pool testing methods

Pool testing methods are typically classified into hierarchical and non-hierarchical methods. In hierarchical methods, individuals are tested in non-overlapping pools at any specific stage of the testing process. The testing plan at any subsequent stage depends on the results of the tests in the previous stage. The Dorfman method is considered a two-stage hierarchical algorithm. Since the Dorfman’s two stage pool testing proposal, several researchers developed extensions of the Dorfman’s original method. These extensions include partitioning pools which test positive, into non-overlapping sub pools repeatedly, until all positive individuals are identified through individual tests. For example, Finucan [27] developed a three-stage pool testing method where initially a master pool that contains all individuals is tested, then sub pools are tested in the middle stage, and finally individual retesting is conducted in the final stage.

Hierarchical pool testing methods are typically called “adaptive” because the test is conducted in stages or rounds and the results of any stage depend on the results of previous stages. These testing methods require a first round of testing to test the pools and a second round of tests for individuals in positive pools. This second round might require extracting samples which could overload laboratories especially if samples are extracted manually. These methods might not be efficient particularly in situations where the results need to be delivered quickly. Although adaptive pool testing methods might require fewer tests, non-hierarchical or “non-adaptive” pool testing schemes; where overlapping pool testing is completed in a single step, allow for parallel testing and do not require extra samples be extracted, which improves the testing efficiency [20, 28].

The array pool testing approach is the most common type of non-hierarchical pool testing algorithms where individual specimens are arranged into rows and columns of an array. Row pools and column pools are simultaneously tested in parallel [29]. In two-dimensional array pool testing algorithms, every individual is typically a member of two pools: one row pool and one column pool such that a sample of each individual is located at the intersection of a unique pair of pools. In the first stage of the testing process, all row pools and all column pools are tested. All individuals who are at the intersection of a positive row pool and a positive column pool need to be retested individually. Under the assumption that tests are error-free, the decision is simple in that all individuals that are at the intersection of a positive row pool and a positive column pool are declared positive [25, 26]. However, under the more realistic assumption that tests might have errors, the decision is more complicated, since it is possible that a row pool tests positives with no column pool testing positive, and the other way around [30].

Typically, tests are subject to errors which can occur for many reasons such as an erroneous testing tool or an inadequate test implementation. Therefore, there is a need to account for these testing errors. Kim et al. [25] developed a two-dimensional pool testing method that takes into consideration testing errors where entire row pools or column pools might be retested. The authors also developed a three-stage pool testing method by adding a master pool and derived models for the expected number of tests for their pool array testing algorithms. Hudgens and Kim [17] analyze the impact of the pool size on the expected number of tests for square array pool testing without master pools and provide bounds for optimal pool sizes in case of homogeneous populations assuming error-free tests.

Kim and Hudgens [18] analyze the performance of three-dimensional array pool testing under the assumption that the population is homogeneous. They find that three-dimensional array pool testing can reduce the expected number of tests compared to two-dimensional array pool testing. However, according to the method of Kim and Hudgens [18], individuals are arranged in three dimensional cubes and the pooling is performed along hyperplanes. This way, every individual becomes a member in three pools but any two hyperplanes will intersect in more than one individual rather than a single individual, which might negatively affect the performance of the algorithm. Mutesa et al. [31] propose an adaptive algorithm for pooling subsamples based on a hypercube structure that, at low prevalence, accurately identifies individuals infected with SARS-CoV-2 using a small number of tests and few rounds of testing.

Haber et al. [32] has reviewed recent developments in pool testing research with a focus on Dorfman’s algorithm for a homogeneous population using several case studies. The authors indicate that most prior research on pool testing focuses on minimizing the expected number of tests and they call for paying more attention to the benefits of pool testing in improving the accuracy of the testing process. The preprint Fargion et al. [33] indicates that for homogeneous populations, array pool testing might yield “mirror” false positives as a result of individuals who are healthy being located at the intersection of a positive row pool and a positive column pool. Yelin et al. [34] report that pool testing can detect COVID-19 infections in pools of up to 64 members. A recent study found that a pool size of five is cost-effective for monitoring the COVID-19 spread at Northeastern University [13].

Recent research analyzes non-adaptive pool testing methods where each individual is assigned to several pools. Hanel and Thurner [35] study the impact of test accuracy on the selection of the pool size with the objective of minimizing the number of tests. They propose to test replicas of the same pool to improve the accuracy on the expense of the efficiency in terms of the number of tests and indicate that no more than two replicas of the same pool improve the testing accuracy while the same pool-replicas of three are worthwhile only in the case of large pool sizes. Another line of research assigns every individual to several pools such that every two individuals are common in at most one pool assuming a homogeneous population and error-free tests [20]. The number of pools to which an individual is assigned is called *pooling* *multiplicity* where all individuals are assigned to an equal number of pools. A multiplicity of *k*, means every individual is a member in exactly *k* pools such that every individual is tested *k* times but in different pools. However, the assumption that tests are error-free is not realistic in many situations. Testing errors on the individual level happen, when an individual specimen who is sick (healthy) is incorrectly declared as negative (positive).

A popular method to detect sick individuals using non-adaptive pool testing is the combinatorial orthogonal matching pursuit (COMP) which is attributed to [36]. According to COMP, any individual in a negative pool is declared definitely healthy while the remaining individuals are considered possibly sick. Since COMP is considered a noiseless pool testing method, hence it produces no ‘false negatives’ but might yield a high rate of ‘false positives’.

The presence of testing errors can introduce false negatives which can be mitigated using the noisy COMP (NCOMP) algorithm. The NCOMP name is attributed to [37] and the basic concept has been introduced by [38, 39]. According to NCOMP, any individual who is a member in a certain minimum number of positive pools is declared sick, otherwise it is declared healthy.

The performance of both COMP and NCOMP is analyzed by [40] who indicate that COMP is a special case of NCOMP. Lets denote the number of pools in which the individual is a member as the membership size (*m*). The authors state that imposing further conditions on the multipooling matrices; other than constant pool size, constant membership size, and dot product between columns of at most one, will not reduce the expected number of false positives in COMP and NCOMP. They also show that increasing the membership size decreases the pooling sensitivity but increases the pooling specificity [40]. A variant of the COMP algorithm is the Definite Defective (DD) algorithm [41] which performs better than COMP in terms of the number of tests in cases when the prevalence level is low [37]. The DD starts by using COMP to identify the definitely healthy. Next, any individual who is the only potentially sick in a positive pool is declared sick, while all other remaining individuals are declared healthy. Since the DD is noiseless, hence it might produce high false negative rates. To overcome the limitations of the DD, a noisy DD algorithm has been developed in [42] in which the test outcomes are based on some pre-specified threshold values. The Noisy DD has been shown to perform better than NCOMP in terms of the number of tests [37] as well as in probability of detection success. However, as indicated earlier, the COMP paradigm is a basic step in other pool testing algorithms and therefore our method is based on the noisy COMP.

Given the growing importance of improving the accuracy of pool testing, recently a smart pool testing software application has been developed based on the Tapestry hybrid pool testing [43] where the COMP is used as an initial stage in the testing process. According to this method, the COMP identifies definitely healthy individuals who are consequently excluded from further investigation. Ghosh et al. [43] excludes not only healthy individuals but also negative pools from further investigation and they analyze the performance of several compressed sensing methods as a second stage of testing following the COMP stage to identify the health status of the remaining possibly sick individuals. In the Tapestry pool testing, each individual is assigned as a member into three pools and any two individuals are common in at most one pool. The test outcomes of individuals are then classified into three classes: sick, healthy, and unidentified [44] and therefore a second round of testing might be needed in rare cases. A hybrid approach is also applied in [45] where a compressed sensing algorithm is used as a second stage of testing after excluding the definitely healthy individuals identified using COMP in the first stage where based on the final testing outcome, individuals are classified as either healthy or sick.

A main difference between our method and Tapestry is that unlike Tapestry in which each individual contributes to exactly three pools, our method is more general since each individual can be a member in any number of pools, up to a certain maximum value as shown by [40], provided that any two individuals are members in exactly one pool. Another limitation of the Tapestry is that it is based on an algorithmically two-stage approach where in the first stage the COMP is applied and then the output of the COMP stage is fed as an input to the CS stage. However, in such two-stage method, errors committed in the first stage are irreversible in the second stage. For example, in cases when the manufacturer’s sensitivity of the test is low then, if the COMP stage erroneously declares a specific individual to be negative, then this individual will not be considered into the second stage the CS stage.

Altman and Bland [46] developed two main measures of testing accuracy: the test sensitivity and the test specificity. The test sensitivity *S*_*e*_ is the proportion of the true positives that are classified correctly by the test while the test specificity *S*_*p*_ is the proportion of the true negatives that are classified correctly by the test [46]. During the pool testing process, the test might be applied on the same sample multiple times whether individually or as a member of a pool. Therefore, the test sensitivity *S*_*e*_ and the test specificity *S*_*p*_ as quoted by the manufacturer are not sufficient to estimate the probability of an individual being correctly diagnosed by the pool testing method. Consequently researchers developed other measures of testing accuracy for pool testing including pooling sensitivity and pooling specificity. The pooling sensitivity *PS*_*e*_ is defined as the probability that an individual is classified as positive by the pool testing algorithm, provided that the individual is sick. While, the pooling specificity *PS*_*p*_ is the probability that an individual is classified as negative by the pool testing algorithm, provided that the individual is healthy [47].

Unlike prior research in pool testing that mainly attempts to minimize the number of tests, this paper aims to improve pool testing accuracy using the same number of tests used by individual testing considering the probability of testing errors and pool multiplicity classification thresholds. This is accomplished by adopting a pooling multiplicity approach where every individual is assigned to several pools such that every two individuals are common in at most one pool. Statistical models are developed to evaluate the impact of pool multiplicity classiffcation thresholds on pool testing accuracy using the receiver operating characteristic (ROC) curve and the area under the curve (AUC).

## 3 Statistical models

Prior research developed several pools formation methods like the Shifted Transversal Pool Testing Design [28] which seeks to reduce the number of joint membership of individuals in any given pool, and at the same time generates pools that intersect in an equal number of locations. These two properties can improve the non-adaptive detection process significantly. A multipool matrix can be generated using the Shifted Transversal Design method, when the pool size is chosen to be a prime number and can be generated using the more general Reed- Solomon method [48] when the pool size is chosen to be a power of a prime (see [40] for detailed illustrations). Given these designs, Schumacher and Tauffer [40] define a multipool as a structure in which all pools are of equal size, every individual has the same membership size (the number of pools in which the individual is a member), and any two pools intersect in at most one location. The authors also prove that a multipool matrix exists if and only if the membership size has an upper bound for the case when the pool size is a prime or a power of a prime. They demonstrate that this upper bound is equal to the pool size plus one, given the pool size is the square root of the population size.

Our method is based on a multipool design [20, 40], where individuals are grouped into pools of size *n* such that every individual is a member in exactly *n* pools and such that any two individuals are common members in exactly one pool. Table 1 provides a list of our model parameters. The *N* individual samples can be arranged in an *n* × *n* square array with the number of rows denoted as *J* and the number of columns denoted as *K* where *J* = *K* for square arrays. Then the pools can be generated by partitioning individuals equally into *J* row pools and also partitioning individuals equally into *K* column pools. For example, Row-Pool(*j*) contains individuals who are located on row *j*. Individuals are marked by their coordinates or location where an individual who is located on the intersection of the *j*^*th*^ row and the *k*^*th*^ column is denoted by *I*_*jk*_. This individual becomes a member in the Row-Pool (*j*) and also a member in Column-pool (*k*). In other words, every individual’s sample is divided into *k* subsamples and assigned to *k* different equally-sized pools of size *n* where no two individuals are common members in more than one pool.

Note that any two pools do not intersect in more than one location if *n* is a prime or a power of a prime [20, 40]. The pools formation process starts by generating *n* patterns of *n* pools each. For example, one pattern could consist of the set of the *J* row pools and another pattern could consists of the set of the *K* column pools. Patterns also can be generated along diagonals where an additional pattern can consist of all the *D* main diagonal pools (running from the upper left corner to the lower-right corner), where *J* = *K* = *D*. More patterns can be generated along other types of diagonals as well [20].

**Fig 1:**
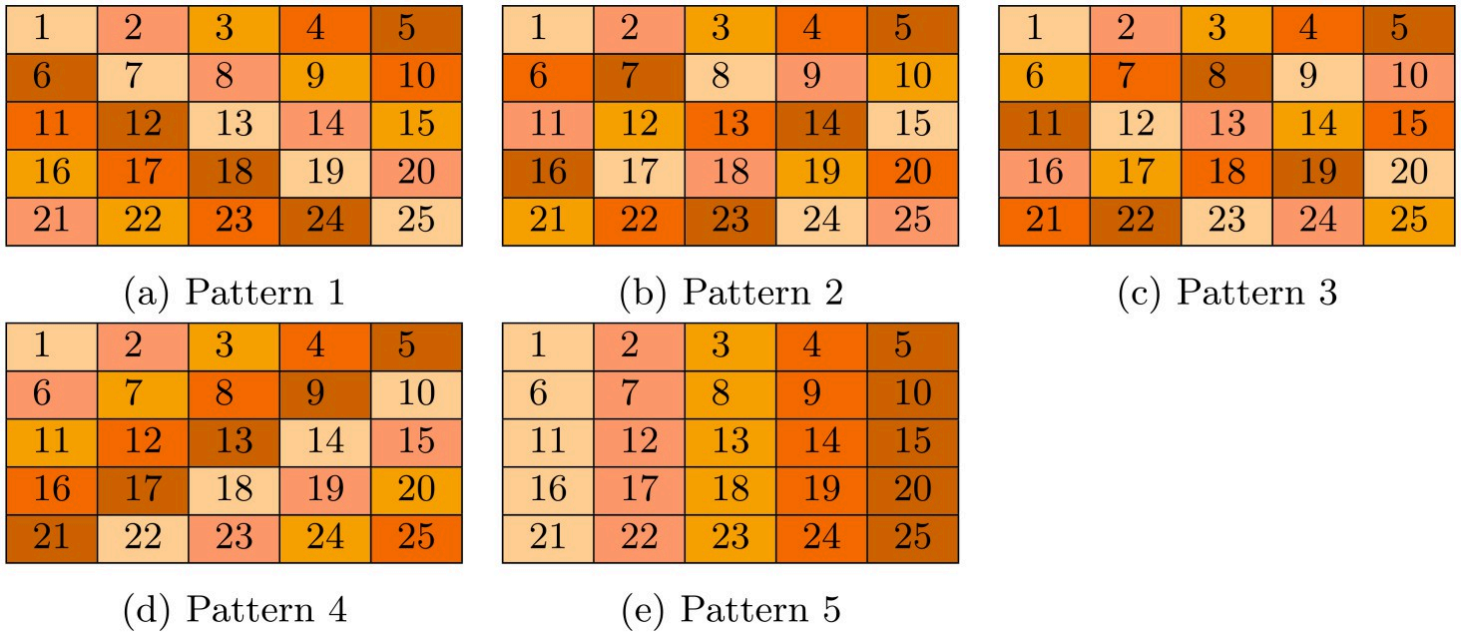
Example 1: An example of 5 patterns with 5 pools each. Every small table represents a pattern, and each color represents a pool in the pattern. (a) Pattern 1, (b) Pattern 2, (c) Pattern 3, (d) Pattern 4, and (e) Pattern 5.

In order to simplify the coding process during simulation, *n* patterns that consist of 5 diagonal patterns, rather than row patterns, column patterns and diagonal patterns, are developed. As an example, lets assume *N* = 25 individuals, hence, $n=J=K=\sqrt{N}=5$. Fig 1(e) shows the diagonal vertical 0-offset (column) pattern with 5 pools where every pool is marked by a distinct color. Compared to prior research, our method has the advantage of reducing the memory requirements significantly since the pool membership data is being calculated by the algorithm rather than storing them as a pooling matrix.

The diagonal patterns are formed by declaring a horizontal offset value (*h*_*offset*) and a vertical offset value (*v*_*offset*) for each pattern. We simplify the process by fixing *v*_*offset* = 1 throughout the pattern and pool formation process. The 1st pool of the 2^nd^ pattern (the 1st diagonal pattern or the main diagonal pattern) is generated using *h*_*offset* = 1; meaning that the first pool in this pattern starts with the upper-right corner individual (*I*_00_) then horizontally we move right by (*h*_*offset* = 1) location and vertically we move down by (*v*_*offset* = 1) location, and so on, until we include *n* individuals into this pool. The 2^nd^ pool in this pattern starts with individual (*I*_01_) and the remaining members of this pool are generated similarly but using *h*_*offset* = 2 and *v*_*offset* = 1. In general, the *i*^*th*^ pool in this pattern will be generated starting with individual (*I*_0*i*_) using *h*_*offset* = 1 and *v*_*offset* = 1. To avoid the “fall-off” (exceeding) the array boundaries, the arithmetic *modulo* *n* function can be used to wrap the pool generation process where the process starts from 0 whenever we reach (*n* − 1) [20]. As in Tauffer (2020), lets represent the members of pool *l* that belongs to pattern *m* as the set *PP*(*l*, *m*) where:

$$\begin{array}{c} PP\left(l,m\right)=\left\{I_{j,(l+j\times m)(mod\;n)}:j=0,1,\cdots ,n-1\right\},\;\forall l,m=0,1,\cdots ,n-1. \end{array}$$

Fig 1(a) shows the 5 pools of the 1^st^ pattern (the 1^st^ diagonal pattern).

Likewise, the 1^st^ pool of the second pattern (the 2^nd^ diagonal pattern) is generated starting with individual (*I*_00_) but with *v*_*offset* = 2, and so on. In general, the *i*^*th*^ pool of the *j*^*th*^ diagonal pattern is generated starting with (*I*_0*i*_) but with *h*_*offset* = *j*. Fig 1(c)–1(e) show the pool formation for the remaining patterns.

Assume a population size of *N* individuals and a multiplicity level $n=\sqrt{N}$ where the *N* individuals can be arranged in an *n* × *n* square array. Each individual’s sample is broken up into *n* sub-samples and the sub-samples are assigned to *n* different pools in *n* different diagonals patterns such that every two individuals are common in at most one pool. Consistent with prior research, it is assumed that:

- *Assumption 1. The true statuses of individuals are independent and identically distributed random variables with probability p of being sick.*
- *Assumption 2. Given that the true status of an individual I_ij_ who is a member of pool P_k_ is sick; i.e., (Y_ij_ = 1), then pool P_k_ tests positive with probability S_e_ and testes negative (i.e. false negative) with a probability 1 − S_e_. This implies that the pool test sensitivity is independent of the pool size.*
- *Assumption 3. Given that all the individuals in pool P_k_ are healthy, then pool P_k_ tests positive (i.e. false positive) with a probability 1 − S_p_ and testes negative (i.e. true negative) with a probability S_p_. This implies that the pool test specificity is independent of the pool size.*
- *Assumption 4. The test outcomes of intersecting pools are conditionally independent of each other.*
- *Assumption 5. The pool size n is a prime number.*

A homogeneous population is assumed and the prevalence is defined as *p* = *P*(*Y* = 1), where *Y*, represents the true status of an individual. Similar to Kim et al. [25], McMahan et al. [26], Aprahamian et al. [49], and Hitt [47] we assume that the true statuses of individuals are mutually independent random variables. Let *X*_*ij*_ = 1 if the test outcome of the individual at the location *ij* is diagnosed positive; *X*_*ij*_ = 0 otherwise. Let *Y*_*ij*_ = 1 if the true status of the individual at the location *ij*, is sick; *Y*_*ij*_ = 0 otherwise.

Let the manufacturer-reported specificity and sensitivity be denoted by *S*_*p*_ = *P*(*X* = 0|*Y* = 0) and *S*_*e*_ = *P*(*X* = 1|*Y* = 1), respectively. Assume that *S*_*e*_ and *S*_*p*_ are known, diagnostic test dependent, independent of the individual’s covariates, independent of the number of individuals per pool, i.e. no dilution. Two main types of testing approaches: individual testing, and pool testing, are compared. Let the individual testing specificity and sensitivity be denoted by *IS*_*p*_ and *IS*_*e*_, respectively, and let the pool testing specificity and sensitivity be denoted by *PS*_*p*_ and *PS*_*e*_, respectively.

**Fig 2:**
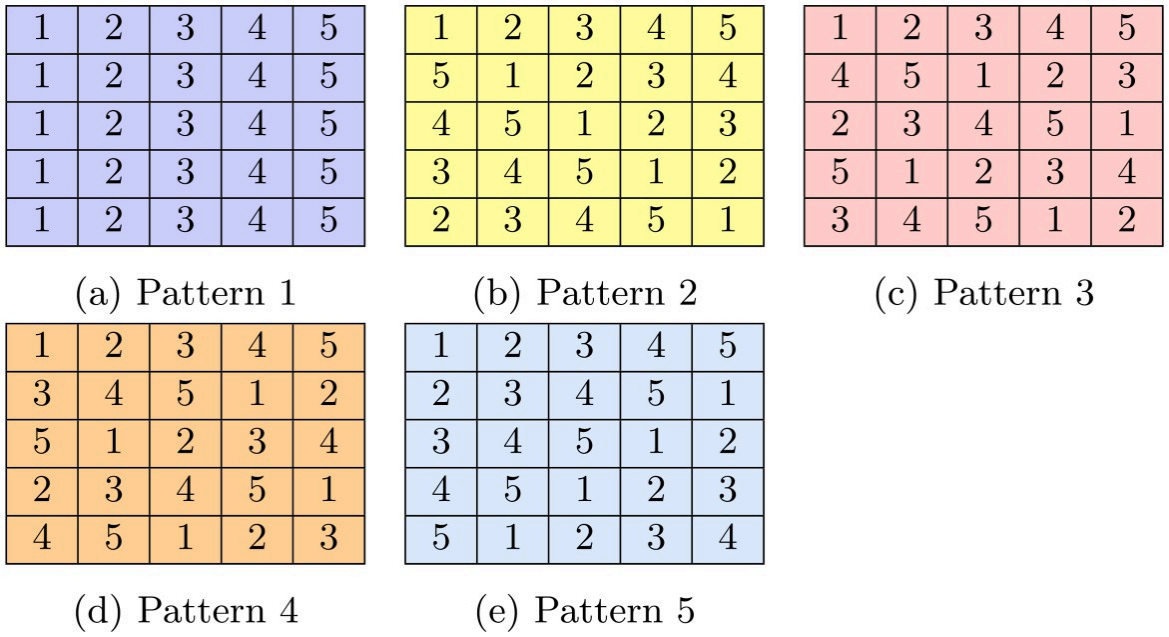
Example 2: An example of a pooling matrix generation process with n = 25. Individual’s positions are fixed in every colored matrix. The numerical value represents the pool number while the color represents the pattern number. (a) Pattern 1, (b) Pattern 2, (c) Pattern 3, (d) Pattern 4, and (e) Pattern 5.

Let *P*_*k*_ represents pool number *k* for *k* = 1, ⋯, *N*. The set of pools to which individual *I*_*ij*_ belongs is denoted as *SP*_*ij*_, i.e.

$$\begin{array}{c} SP_{ij}=\left\{P_{k}:I_{ij}\in P_{k},k=1,\cdots ,N\right\} \end{array}$$

for every pattern *l* = 0, ⋯, *n* − 1,

$$\begin{array}{c} k=((j-(i\times l))modn)+1+l\times n \end{array}$$

where *i* = 0, ⋯, *n* − 1 is the row number and *j* = 0, ⋯, *n* − 1 is the column number of the location of individual *I*_*ij*_. Every individual *I*_*ij*_ belongs to exactly *n* pools. Pools are arranged in *n* patterns of *n* pools each. Rather than storing the pool information as a binary pooling matrix, our algorithm assigns individuals to pools at run-time as can be seen from Example 2 in Fig 2 below. This feature has the advantage of saving memory considerably.

Throughout this paper, the terms “positive” and “negative” are used to refer to the test outcomes (i.e., to indicate the presence or absence of the disease based on the test outcomes, respectively), while we use the terms “sick” and “healthy” to indicate the “true status” of an individual. To simplify the presentation, the term “individual” is used to refer both to the individual and to the sample taken from the individual.

**Table 1:** The model parameters.

Parameter	Description
*N*	The population size
*n*	The pool size, $n=\sqrt{N}$
*p*	The incidence of the infection
*I*_*ij*_	The individual who is in the *i*^*th*^ row and the *j*^*th*^ column
*X*_*ij*_	The test outcome of individual *I*_*ij*_
*Y*_*ij*_	The true status of Individual *I*_*ij*_
*P*_*k*_	The pool number *k*, for *k* = 1, ⋯, *N*
*XP*_*k*_	The test outcome of pool *P*_*k*_
*SP*_*ij*_	The set of pools to which individual *I*_*ij*_ belongs
0 ≤ *S*_*e*_ ≤ 1	Test sensitivity
0 ≤ *S*_*p*_ ≤ 1	Test specificity
*IS*_*p*_	Individual testing specificity
*IS*_*e*_	Individual testing sensitivity
$PS_{p}\left(T\right)$	Pool testing specificity for a threshold of T=1,⋯,n.
$PS_{e}\left(T\right)$	Pool testing sensitivity for a threshold of T=1,⋯,n.

In the multiplicity pool testing method (*MPTM*) we define *n* different classification protocols to identify positive individuals. Each protocol identifies positive individuals based on a minimum threshold value representing the number of positive pools in which that individual is a member. In particular, protocol *i* indicates that an individual will be declared positive if the test outcome of at least *i* of its pools turn positive (classification threshold value of *i*). The multiplicity pool testing sensitivity with a threshold of T assuming a homogeneous population has been derived by [40] as follows:

Let $n=\sqrt{N}$, where *n* is a prime number, be the multiplicity level and let T be the classification threshold, where T=1,⋯,n, then the multiplicity pool testing sensitivity $PS_{e}\left(T\right)$ can be expressed as

PSe(T)=∑i=Tn(ni)(1-(1-Se)(Sp(1-pSe)n-1))i((1-Se)(Sp(1-pSe)n-1))n-i (1)

for any values of *S*_*e*_, *n*, and T.

According to the proposed multiplicity pool testing method, an individual who is in the *i*^*th*^ row an the *j*^*th*^ column is declared positive (i.e. *X*_*ij*_ = 1) if at least T of its pools test positive for any specific classification threshold T=1,⋯,n.

More formally, for individual *I*_*ij*_,

$$\begin{array}{c} if\left(\sum_{k=1:P_{k}\in SP_{ij}}^{N}XP_{k}\right)\geq T),\;\text{then}\;X_{ij}=1, \end{array}$$

where *i* = 0, ⋯, *n* − 1; *j* = 0, ⋯, *n* − 1; and *k* = 1, ⋯, *N*,

The multiplicity pool testing specificity with a threshold of T assuming a homogeneous population has also been derived by [40] as follows:

Let $n=\sqrt{N}$, where *n* is a prime number, be the multiplicity level and let T be the classification threshold, where T=1,⋯,n, then the Multiplicity pool testing specificity $PS_{p}\left(T\right)$ can be expressed as

PSp(T)=1-∑i=Tn(ni)(1-(Sp(1-pSe)n-1))i(Sp(1-pSe)n-1)n-i (2)

for any values of *p*, *S*_*e*_, and *S*_*p*_, *n*, and T.

**Fig 3:**
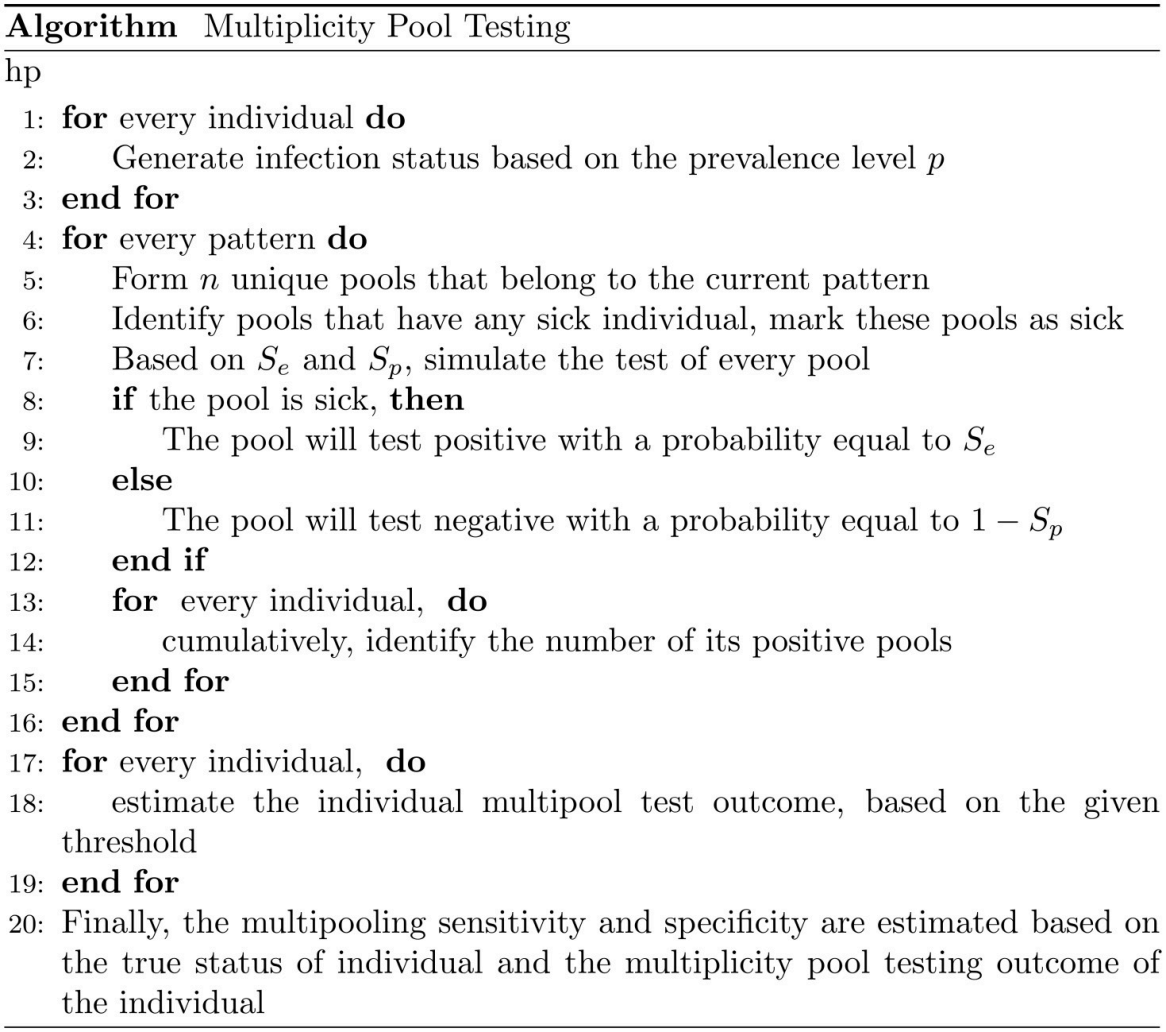
Multiplicity pool testing algorithm.

The outline of the multiplicity pool testing algorithm is presented in Fig 3 below. The R code implementation of the algorithm is abailable at https://github.com/ralsehib/Multiplicity-Pool-Testing/blob/main/Multiplicity%20Pool%20Testing.R. Our R code implementation has the advantage of being concise as well as supporting parallelism. The code generates pools at run-time rather than storing the pool information as a binary matrix which saves memory significantly. The R software package use is RStudio version 1.1.419.

### 3.1 The area under the ROC curve (AUC)

The impact of the pool testing conditions on the joint accuracy measures (pool testing sensitivity and pool testing specificity) of classification in diagnostic settings can be analyzed using the receiver operating characteristic (*ROC*) curve which is a commonly used visual illustration. ROC curves display the true positive rates versus the false-positive rates for a range of classification threshold values. The ROC curve describes the ability of the test to identify sick from healthy individuals and it can also be used in identifying the threshold value that gives the optimal testing accuracy [50]. The ROC curve is a plot of *sensitivity* versus (1 − *specificity*) for a range of possible classification threshold values and it represents a trade-off between sensitivity and specificity.

An ROC curve starts at the (0, 0) coordinate, corresponding to the case where all test results are negative and ends at the (1, 1) coordinate, corresponding to the case where all test results are positive. The typical lower limit of the ROC curve is a diagonal line that connects the lower left and the upper right corners of the graph with an area under the curve of 0.5. In other words, the diagonal line that connects the (0, 0) and (1, 1) points represents the ROC curve of a random test that does not distinguish sick from healthy individuals. ROC curves that lie above this diagonal has some diagnostic ability where the farther the ROC curve from the diagonal (the closer to the upper left-hand corner), the better the diagnostic accuracy of the test [50, 51].

A popular measure of test accuracy is the area under the ROC curve, denoted as (AUC) [51]. The AUC is calculated using the trapezoidal rule. Denote the coordinate of the curve given the threshold *i* as (*x*_*i*_, *y*_*i*_) ∀*i* = 1, ⋯, *n*. Let the initial coordinate of the ROC curves be always (0, 0). Note that,

$$\begin{array}{c} x_{i}=1-PS_{p}\left(i\right)\;\forall i=1,\cdots ,n \end{array}$$

and,

$$\begin{array}{c} y_{i}=PS_{e}\left(i\right)\;\forall i=1,\cdots ,n \end{array}$$

Hence, the total area under the curve (*TAUC*) can be expressed as:

$$\begin{array}{c} TAUC=\sum_{i=1}^{n}\left(\left(\left(1-PS_{p}\left(i+1\right)\right)-\left(1-PS_{p}\left(i\right)\right)\right)\times PS_{e}\left(i\right)\right)\\ +(\frac{1}{2}\times \left(\left(1-PS_{p}\left(i+1\right)\right)-\left(1-PS_{p}\left(i\right)\right)\right)\times \left(PS_{e}\left(i\right)-PS_{e}\left(i+1\right)\right)) \end{array}$$

## 4 Results and discussion

The performance of the multiplicity pool testing is evaluated and the overall testing accuracy is estimated through simulation using the *R* software package. The simulation code is efficiently developed by considering *n* diagonal patterns, rather than row, column, and diagonal patterns. The true status of individuals will be randomly generated based on a Bernoulli distribution with the prevalence level of the disease *p* as a given probability parameter. The simulation of our method generates a “sick” true status with a probability of *p* and generates a “healthy” true status with probability 1 − *p*. Individual test outcomes are estimated based on a Bernoulli distribution with the manufacturer testing sensitivity *S*_*e*_ or the manufacturer testing specificity *S*_*p*_ as given probability parameters.

After estimating the test outcomes of all individuals through pool testing, the values of the accuracy measures are calculated in a way that is similar to that of individual testing explained above. For both individual testing and pool testing simulations, we run 1000 independent repetitions to take variability into consideration where averages across the 1000 repetitions are reported.

### 4.1 Accuracy measures vs. prevalence

Assume a population of *N* = 25, then 25 pools are formed where each pool contains $\sqrt{N}=5$ members. Every individual will be a member in exactly 5 different pools in five different patterns (i.e. a zero step-based diagonal pool (column pool), a one step-based diagonal pool, a two step-based diagonal pool, a three step-based diagonal pool, and a four step-based diagonal pool).

**Fig 4:**
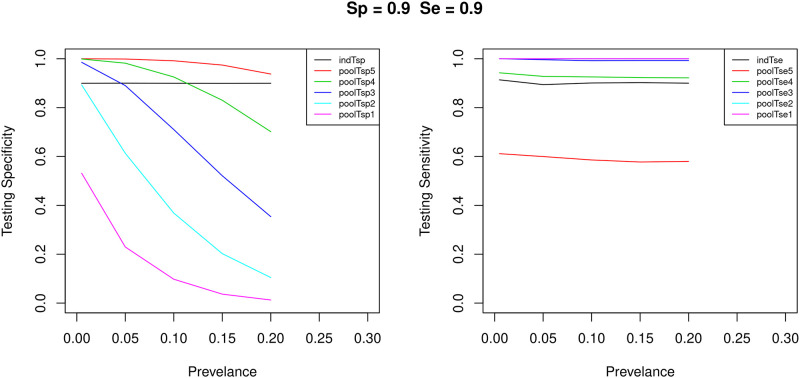
Comparison of the test accuracy measures: Specificity and sensitivity, for individual testing and pool testing.

**Fig 5:**
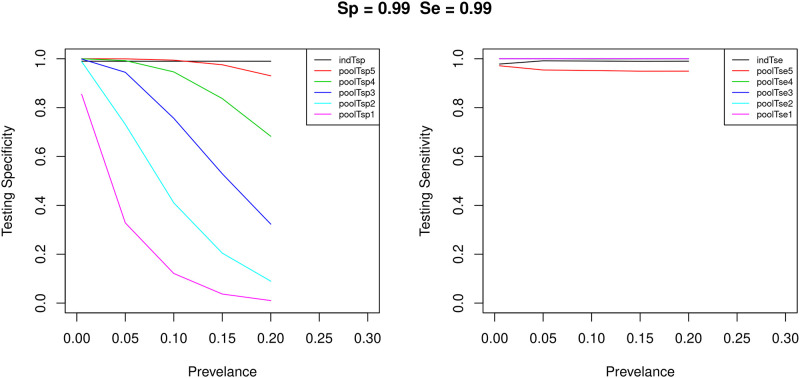
Comparison of the test accuracy measures: Specificity and sensitivity, for individual testing and pool testing.

Let’s assume 5 different levels of prevalence ranging between 0.005 and 0.20, as well as 3 different values of *S*_*p*_ and *S*_*e*_ ranging from 0.90 to 0.99. The pool testing multiplicity level is assumed constant with a value of 5 throughout the first stage of the simulation. Comparison of the multiplicity pool testing and the individual test accuracy measures: specificity and sensitivity, versus different values of prevalence between 0.005 and 0.20 for individual testing and pool testing are shown in Figs 4 and 5. The left figure presents the testing specificity and the right figure presents the testing sensitivity. The solid line represents the test accuracy measures. Different colors of curves represent different pool testing classification thresholds. The black color represents individual testing. The red color represents pool testing with a threshold of 5. The green color represents pool testing with a threshold of 4. The blue color represents pool testing with a threshold of 3. The light-blue color represents pool testing with a threshold of 2. The pink color represents pool testing with a threshold of 1.

From the experimental results, it can be concluded that under certain conditions, the, multiplicity pool testing method gives higher accuracy compared to individual testing without additional cost. For example, when the prevalence level is low; e.g, *p* ≤ 0.1, classification threshold 4 gives higher pool testing sensitivity and higher pool testing specificity compared to individual testing (manufacture reported sensitivity and specificity). This is particularly true for the case when the manufacture reported sensitivity and the manufacturer reported specificity are low; i.e. *S*_*e*_ = 0.9 and *S*_*p*_ = 0.9. Even for the case when the manufacture reported sensitivity and the manufacturer reported specificity are high; i.e. *S*_*e*_ = 0.99 and *S*_*p*_ = 0.99, classification threshold 4 gives higher pool testing sensitivity and higher pool testing specificity compared to individual testing, but only when the prevalence level is ≤0.05.

The benefit gained in accuracy is higher for the case when the prevalence level is low and the manufacturer reported specificity and sensitivity are low. For example, for *p* = 0.050, from Fig 4, when *S*_*e*_ = 0.90 and *S*_*p*_ = 0.90 a threshold of 1 yields an improvement gain in testing sensitivity of pool testing over individual testing of about 11.9% compared to an improvement gain of 0.8% when *S*_*e*_ = 0.99 and *S*_*p*_ = 0.99. The simulation results show that when the prevalence is high and the test tool manufacturer’s reported accuracy is high then there is no need to use pool testing to improve accuracy because under these conditions the individual accuracy is higher than the pool testing accuracy.

Typically, false negatives might lead to significant risky consequences compared to false positives since false positives could be subject to further verification testing [52, 53]. These consequences include worsening medical complications of the infected individual and the continuous spread of the disease, especially if the individual has many contacts. Therefore, there is a paramount need to develop testing methods that mainly reduce the probability of false negatives as a main objective and at the same time reduce the probability of false positives as a secondary objective. The probability of false negative where a small number of sick individuals are missed, is associated with a high value of test sensitivity [54, 55]. In other words, the probability of false negative is inversely proportional to the test sensitivity. The results show that different classification thresholds give different levels of pool testing accuracy depending on the pool testing conditions. For example, Fig 4 shows that, for prevalence level of *p* = 0.005, classification threshold 4 gives higher pool testing sensitivity and higher pool testing specificity compared to individual testing. However, if perfect pool testing sensitivity; i.e. *PS*_*e*_ of 1 is required, then classification threshold 3 could be chosen even if its pool testing specificity is less than that of threshold 4, since it still gives higher pool testing specificity compared to individual testing.

### 4.2 Classification accuracy

**Fig 6:**
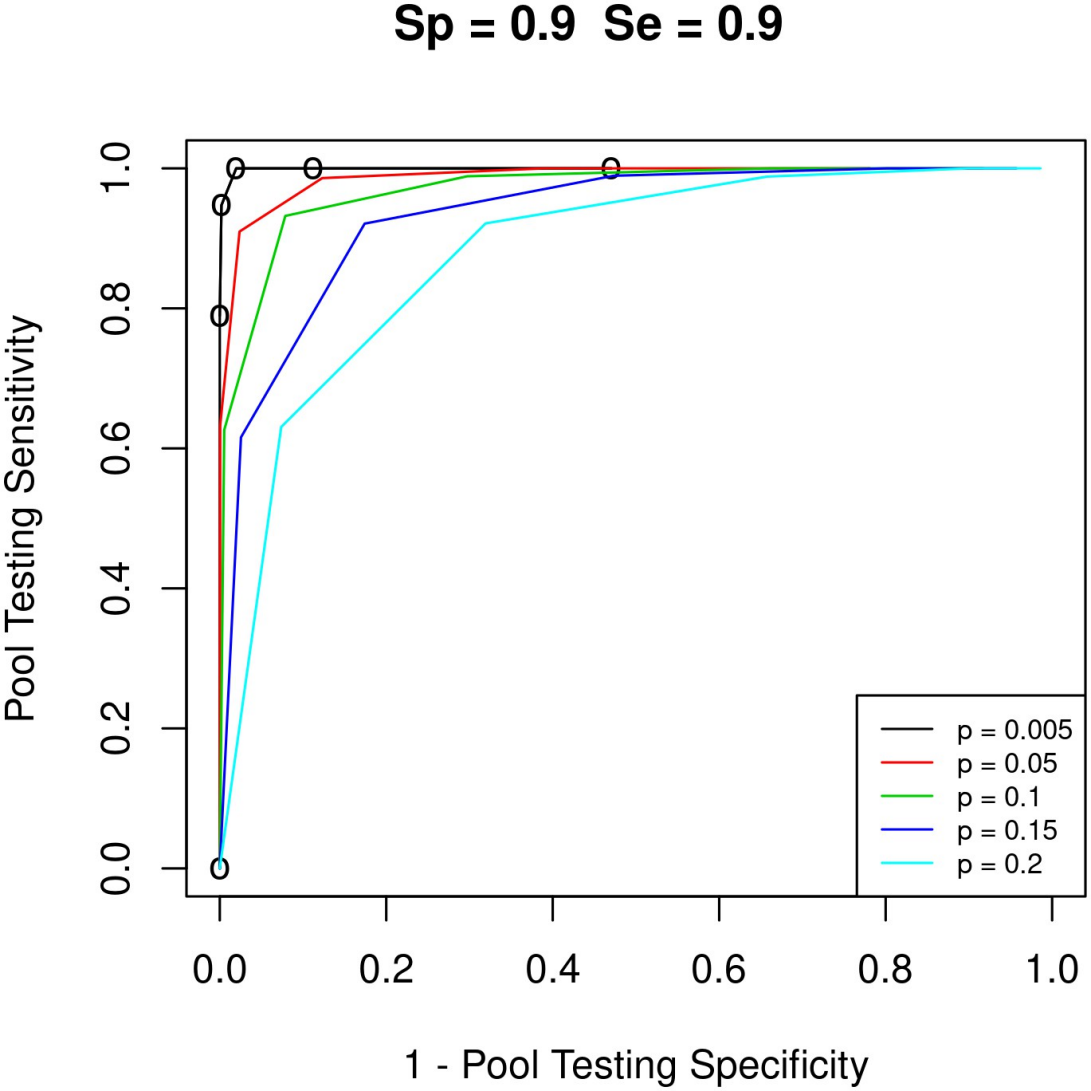
ROC curve for several prevalence levels given *S*_*p*_ = 0.90 and *S*_*e*_ = 0.90.

In the ROC curve we plot the (1 − *specificity*) on the x-axis and the sensitivity on the y-axis where each line on the plot represents a different prevalence level *p*. The performance of the pool testing method is simulated for a population of 25 individuals with a multiplicity level of 5 using different threshold values and different testing conditions. To examine the impact of different levels of prevalence on the classification accuracy, we let *p* = 0.005, 0.0.5, 0.1, 0.15, and 0.2. For each *p*, we experiment with different values of the manufacturer-reported specificity *S*_*p*_ and sensitivity *S*_*e*_, where we let the values of *S*_*p*_ and *S*_*e*_ = 0.90, 0.95, and 0.99 resulting in 9 different combinations of testing accuracy measures. Therefore, we get 9 graphs with each graph displaying 5 ROC curves. These curves are plotted using five classification threshold values T=1,2,⋯,5. Given the values of *p*, *S*_*p*_, and *S*_*e*_, the ROC curves enable us to identify the classification threshold value that should be employed to get the optimal testing accuracy (the highest true positive rate and at the same time the lowest false positive rate). Fig 6 shows the ROC curves under several testing conditions.

The experimental results show that different pool testing conditions (e.g. prevalence, *S*_*e*_, and *S*_*p*_) might require different classification thresholds to obtain the best pool testing accuracy. For example, in the case of a population of 25, a manufacturer reported specificity (*S*_*p*_ = 0.90), manufacturer reported sensitivity (*S*_*e*_ = 0.90), and a prevalence (*p* = 0.005), pool testing sensitivity of 1 can be achieved for several threshold values. From Fig 6, we can see that the false positive rate in pool testing for the threshold value of 3 is approximately 2% where from the same figure we can see that when the prevalence is 0.1, the false positive rate is equal to 66% which is achieved using a threshold value of 2, where in both cases, the pool testing sensitivity is 1. This example shows that the classification threshold should be selected cleverly to obtain the highest testing accuracy.

For a batch size of 25, from the ROCs in Fig 6 we observe that as the prevalence value decreases the pool testing performance in terms of accuracy, as expected increases. Also, from this figure, we observe that, as expected, as the manufacturer reported accuracy increases, the accuracy of the pool testing method improves, as measured by the ROCs, for the different levels of prevalence. Also, we observe that different pool testing methods yield different testing accuracy levels depending on the testing conditions i.e. prevalence, manufacturer reported specificity, manufacturer reported sensitivity and the threshold value. Therefore, there is a need to develop a software tool or an application to associate the different threshold values with the testing conditions in order to identify the classification thresholds that give the highest performance in terms of accuracy.

### 4.3 Impact of the manufacturer’s sensitivity and specificity on the AUC

**Fig 7:**
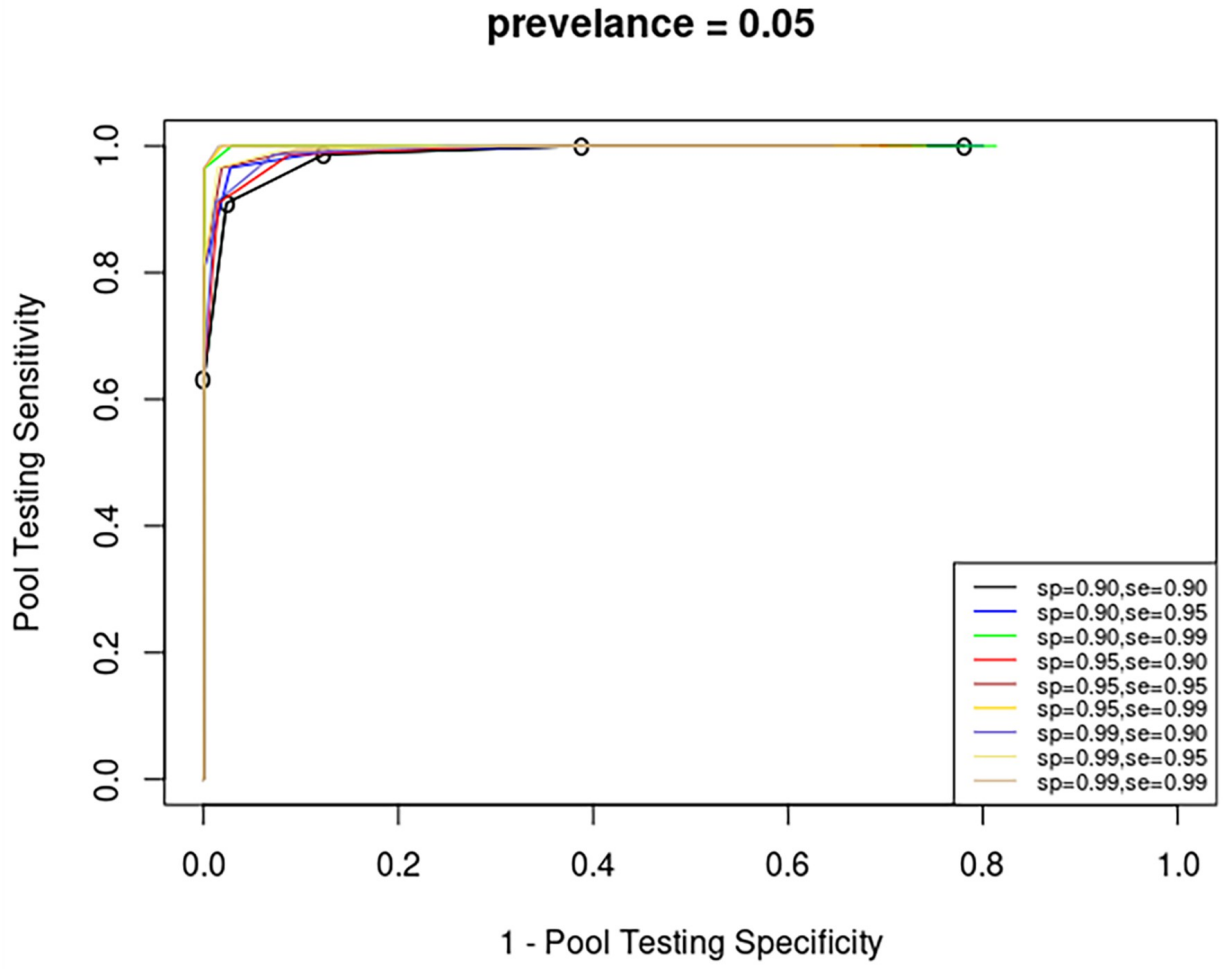
ROC curve for several manufacturer testing specificity and sensitivity levels given *p* = 0.05.

**Fig 8:**
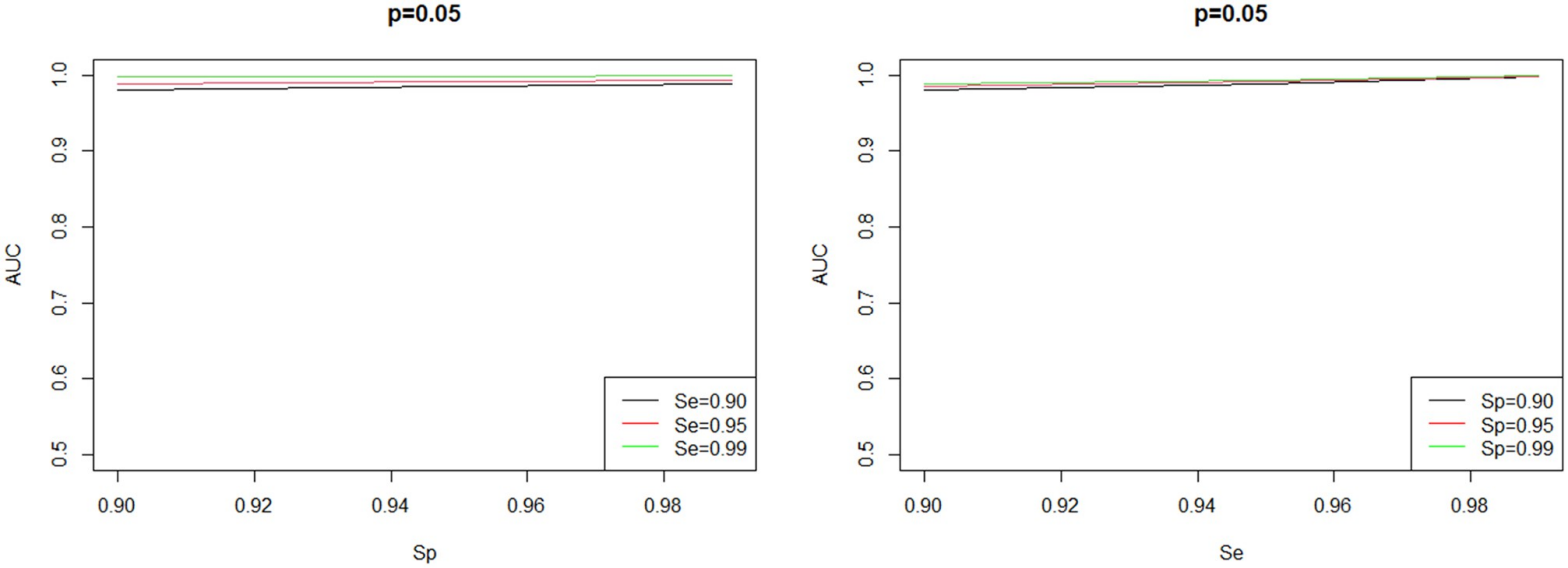
The improvement in the pool testing accuracy, measured by the AUC as a function in *S*_*e*_ and *S*_*p*_.

The AUC for full multiplicity pool testing using nine tests with different values of manufacturer’s test sensitivity and specificity for five different levels of prevalence is shown on Fig 7. The figure shows that for a fixed value of manufacturer’s test sensitivity, the AUCs of the different tests are almost similar to each other. Typically, higher manufacturer’s test sensitivity and manufacturer’s test specificity incurs higher cost. The findings show that significant cost savings can be earned through multiplicity pool testing using low–cost tests. For example, Fig 8 shows that the improvement in the pool testing accuracy, measured by the AUC, in the case of prevalence level of 0.05. From the figure, it is clear that using a low-cost test yields accuracy that is comparable to a high cost-test.

In other words, using a test of low manufacturer’s specificity might incur lower testing costs and at the same time gives comparable pool testing accuracy to other higher-cost tests. For example, from Fig 7, for a prevalence level of *p* = 0.05, using a test of manufacturer’s sensitivity and specificity of *S*_*e*_ = 0.90 and *S*_*p*_ = 0.90, respectively gives an AUC of 0.980 while using a test of manufacturer’s sensitivity and specificity of *S*_*e*_ = 0.9 and *S*_*p*_ = 0.99, respectively gives an AUC of 0.988 Note that the percentage of gain in accuracy is less than 0.82% On the other hand, from Fig 7, for a prevalence level of *p* = 0.05, using a test of manufacturer’s sensitivity and specificity of *S*_*e*_ = 0.90 and *S*_*p*_ = 0.90, respectively gives an AUC of 0.980 while using a test of manufacturer’s sensitivity and specificity of *S*_*e*_ = 0.99 and *S*_*p*_ = 0.90, respectively gives an AUC of 0.998 Note that the percentage of gain in accuracy is about 1.8%. Therefore, as can be seen from the figure, a low-cost test leads to accuracy that is comparable to a high cost-test. However, the manufacturer’s test sensitivity has more significant impact on the accuracy of pool testing compared to that of manufacturer’s test specificity. In other words, from multiplicity pool testing perspective, if the test cost is a critical factor in selecting a certain type of test (among tests of the same manufacturer’s test sensitivity), then a test of lower manufacturer’s test specificity might be an optimal option.

### 4.4 The impact of the batch size on the AUC

A set of *N* individuals can be partitioned into batches of different sizes before applying pool testing. For example, a set of 100 individuals can be divided into 4 batches of size of 25 individuals each or can be divided into 25 batches of size of 4 individuals each, where pool testing can be conducted on each batch. We analyze the impact of different batch sizes on the pool testing specificity, by considering different batch sizes and different prevalence levels.

The performance of the diagnostic test can be evaluated by estimating the area under the ROC curve (*AUC*). The AUC takes values between 0 and 1 and AUCs that have values close to 1 indicate high testing accuracy. Once the ROC curves are generated, the AUC for every curve can be estimated using either the Trapezoidal rule or the Simpson’s rule. In this paper, we use the Trapezoidal rule since the generated curves are not smooth curves because they are developed mainly by connecting several points with straight lines.

**Fig 9:**
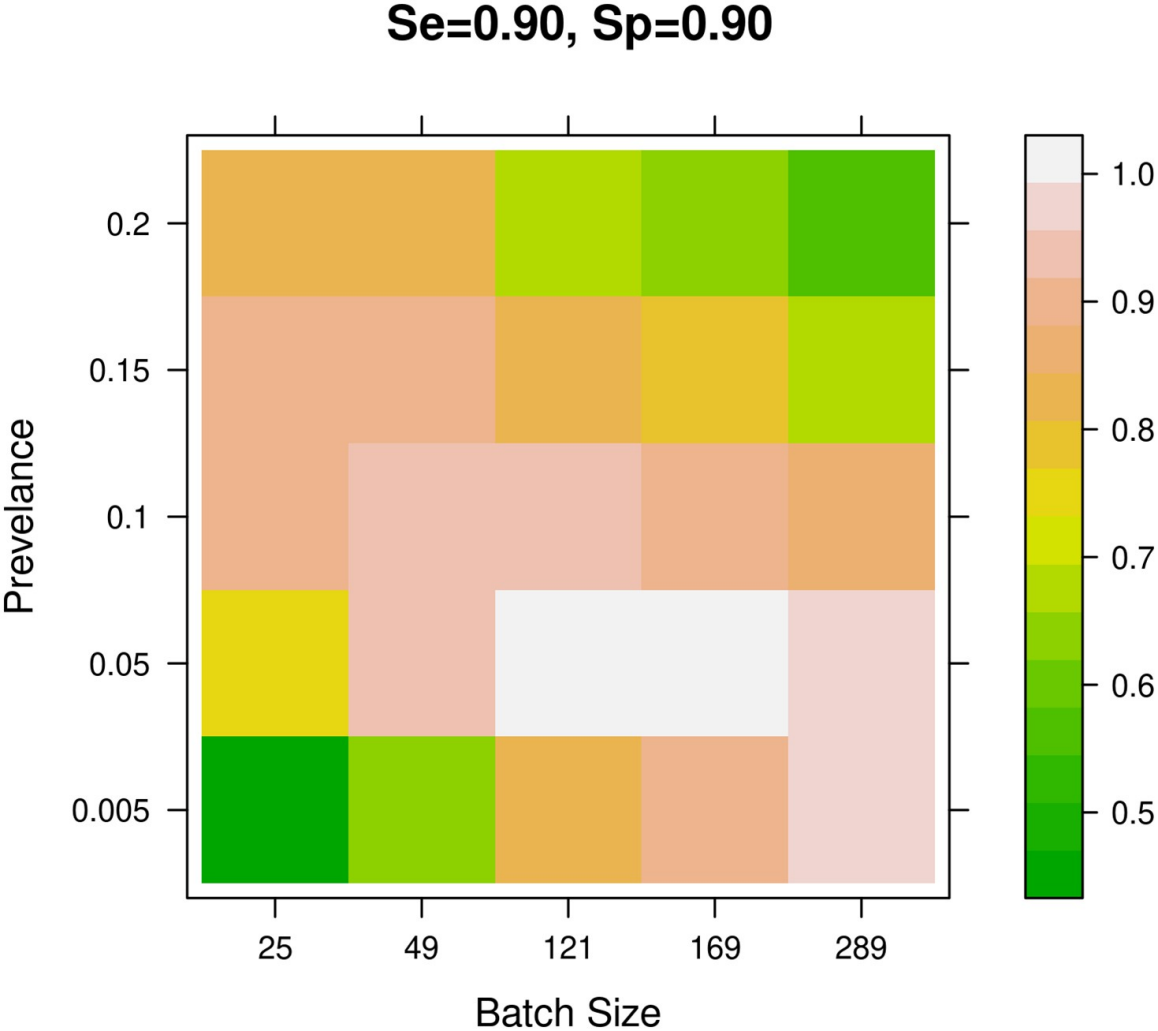
AUC heat map for *Sp* = 0.90 and *Se* = 0.90.

The estimated AUCs are visually displayed using color-coded heat maps to represent the pool testing accuracy given different prevalence levels and batch sizes. Fig 9 shows the heat maps of the AUC for each combination of the manufacturer’s reported sensitivity of 0.90, 0.95, and 0.99 and the manufacturer’s reported specificity of 0.90, 0.95, and 0.99. Observe that from Fig 9 there is a banana-shaped pattern representing the performance of different batch sizes under different levels of prevalence. As can be seen from Fig 9, for low prevalence levels, pool testing using large batch sizes has higher accuracy than pool testing using small batch sizes. While for high prevalence levels, pool testing using small batch sizes performs better than pool testing using large batch sizes.

For every combination of the manufacturer’s reported sensitivity and the manufacturer’s reported specificity, the AUC results can be used as a guide for selecting the recommended batch size for every given prevalence value. Fig 9 shows the heat maps of the AUC for each combination of the manufacturer’s reported sensitivity of 0.90, 0.95, and 0.99 and the manufacturer’s reported specificity of 0.90, 0.95, and 0.99. A future research direction is to develop a software tool or an application to associate the different batch sizes with the testing conditions in order to identify the batch size that gives the highest performance in terms of accuracy.

### 4.5 Implications

This research has demonstrated that pool testing can be used to improve the testing accuracy (i.e. testing sensitivity as well as testing specificity). In particular, it is demonstrated that under certain conditions, the multiplicity pool testing performs better compared to individual testing in terms of testing accuracy without the need for extra tests. Furthermore, the impact of several classification threshold values on the testing accuracy is analyzed. For instance, a naive pool testing algorithm might use a threshold value of 1 in all different conditions whereas the proposed approach enables decision makers to identify under what conditions to get higher testing sensitivity (higher true positive rate) and at the same time higher testing specificity (lower false positive rate). For example, for a population of size 25, *S*_*p*_ = 0.90, *S*_*e*_ = 0.90, and the prevalence *p* = 0.005, Fig 6 shows that a naive approach might choose a threshold value of 1, which would give the highest pool testing sensitivity *PS*_*e*_ = 1, but would give a false positive rate (*FPR*) = 47%, while a smarter approach would recommend a threshold value of 3 which will give us the same pooling sensitivity *PS*_*e*_ = 1, but with much lower *FPR* of 1.9%. Additionally, the results indicate that different batch sizes can be used intelligently, depending on the prevalence level of the disease, to improve the performance of the pool testing method.

## 5 Future work

The independence assumptions simplify the modelling process but might not be realistic in real testing situations since contamination due to handling errors before pooling might increase the false positive rate. Therefore, future research can relax the independence assumption since lab handling errors might affect several pools concurrently.

A growing line of research has started to investigate the impact of dilution on pool testing accuracy especially for large pool sizes since dilution might increase the rate of false negatives. For example, when applying Tapestry to real testing situations, the authors in [44] conducted three real experiments. In the first one, they accounted for dilution by increasing the pooled amount of a positive sample. They indicated however, that the impact of dilution was not significant and therefore in the other two experiments, they pooled equal amounts of samples regardless of whether the individual was healthy or sick.

A recent study reported a pooling sensitivity of 93%, 91%, and 81% for pools of size, 5, 10, and 50 respectively, using a PCR test with 99% manufacturer reported sensitivity for individual tests. The authors suggest that pool testing could be used mainly for the screening of asymptomatic individuals [56]. Another study which used pool testing for the screening of 7400 healthcare workers, revealed that in situations of low prevalence levels, dilution as a result of pooling did not yield significant loss in testing sensitivity [57]. A contemporary study proposes to use swab pooling in which pools are formed at the time of sample collection. Under this scheme, two swabs are collected from every individual such that the first is stored in an individual tube and the other is inserted in a pool with a size of up to 16 different samples, collected individually within a period of one hour. The study focused on asymptomatic individuals in a low-prevalence setting where authors report that the dilution impact was insignificant since swab pooling and individual testing delivered highly similar performance in terms of diagnostic accuracy [58]. Therefore, there is a need for future research to analyze the impact of dilution on the multiplicity pool testing process.

## 6 Conclusion

This paper investigates the impact of pooling multiplicity on the accuracy of pool testing by developing models for higher levels of multiplicity pool testing taking the probability of testing errors into consideration. Through simulation, the impact of several positivity classiffcation protocols (thresholds) on pool testing accuracy: specificity and sensitivity, is evaluated using the ROC and the AUC. In addition, the impact of the batch size on the pool testing accuracy is also examined. The results indicate that under certain conditions multiplicity pool testing yields superior testing accuracy compared to individual testing without additional cost. The findings also demonstrate that pool testing gives higher gains in terms of pool testing sensitivity compared to individual testing in the case when the manufacturer reported sensitivity and the prevalence are low. The findings also reveal that the improvement in accuracy is a function in the multiplicity level, the classification threshold, and the batch size where the performance can be improved using a batch size that is inversely proportional to the prevalence level. Moreover, the results indicate that multiplicity pool testing can reduce the total cost of the testing process since under multiplicity pool testing. The manufacturer’s test sensitivity however has more significant impact on the accuracy of pool testing compared to that of manufacturer’s test specificity.
