# Correction: Statistical modeling and evaluation of the impact of multiplicity classification thresholds on the COVID-19 pool testing accuracy

**DOI:** 10.1371/journal.pone.0307053

**Published:** 2024-07-09

**Authors:** Omar De La Cruz Cabrera, Razan Alsehibani

An additional affiliation is missing for the second author. Razan Alsehibani is also affiliated with Department of Statistics and Operations Research, King Saud University, Riyadh, Kingdom of Saudi Arabia.
